# Association Between Psychological Distress, Cognitive Complaints, and Neuropsychological Status After a Severe COVID-19 Episode: A Cross-Sectional Study

**DOI:** 10.3389/fpsyt.2021.725861

**Published:** 2021-09-03

**Authors:** Clément Gouraud, Hugo Bottemanne, Khadija Lahlou-Laforêt, Anne Blanchard, Sven Günther, Salma El Batti, Edouard Auclin, Frédéric Limosin, Jean-Sébastien Hulot, David Lebeaux, Cédric Lemogne

**Affiliations:** ^1^Université de Paris, AP-HP, Hôpital Hôtel-Dieu, DMU Psychiatrie et Addictologie, Service de Psychiatrie de l'adulte, Paris, France; ^2^Sorbonne University, AP-HP, Pitié-Salpêtrière Hospital, DMU Neurosciences, Service de Psychiatrie de l'adulte, Paris Brain Institute – Institut du Cerveau (ICM), UMR 7225 / UMR_S 1127, / CNRS / INSERM, Paris, France; ^3^Université de Paris, AP-HP, Hôpital Européen-Georges Pompidou, DMU Psychiatrie et Addictologie, Service de Psychiatrie de l'adulte, Paris, France; ^4^CIC1418 and DMU CARTE, AP-HP, Hôpital Européen Georges-Pompidou, Paris, France; ^5^Université de Paris, Innovative Therapies in Haemostasis, INSERM, Paris, France; ^6^Service de Physiologie, AH-HP, Georges Pompidou European Hospital, Paris, France; ^7^Université de Paris, Service de Chirurgie Vasculaire, AP-HP, Hôpital Européen Georges Pompidou, 20 rue Leblanc, Paris, France; ^8^Université de Paris, Service d'oncologie médicale, AP-HP, Hôpital Européen Georges Pompidou, 20 rue Leblanc, Paris, France; ^9^Université de Paris, AP-HP, Hôpital Corentin-Celton, DMU Psychiatrie et Addictologie, Service de Psychiatrie de l'adulte et du sujet âgé, INSERM, Institut de Psychiatrie et Neurosciences de Paris (IPNP), UMR_S1266, Paris, France; ^10^Université de Paris, Service de Microbiologie, Unité Mobile d'Infectiologie, AP-HP, Hôpital Européen Georges Pompidou, 20 rue Leblanc, Paris, France; ^11^Université de Paris, AP-HP, Hôpital Hôtel-Dieu, DMU Psychiatrie et Addictologie, Service de Psychiatrie de l'adulte, INSERM, Institut de Psychiatrie et Neurosciences de Paris (IPNP), UMR_S1266, Paris, France

**Keywords:** anxiety, attention, comorbidity, concentration, COVID-19, depression, long COVID, psychiatric disorder

## Abstract

**Background:** Cognitive complaints are frequent after COVID-19 but their clinical determinants are poorly understood. This study aimed to explore the associations of objective cognitive performances and psychological distress with cognitive complaints in COVID-19 survivors.

**Materials and Methods:** Patients previously hospitalized for COVID-19 in a university hospital during the first wave of COVID-19 pandemic in France were followed-up at 1 month after their admission. Cognitive complaints were self-reported and standardized instruments were used to assess neuropsychological status (Digit Symbol Substitution Test, Semantic Verbal Fluency Test, Mini Mental Status Examination) and psychological distress (Hospital Anxiety and Depression Scale, HADS). Multivariable analyses were adjusted for age, sex, admission in intensive care unit (ICU) and need for oxygen and C-reactive protein.

**Results:** One hundred patients (34% women, median age: 60 years [interquartile range: 49–72)] completed the neuropsychological assessment at follow-up. In multivariable analyses, cognitive complaints at 1-month were associated with greater HADS score (OR for one interquartile range: OR: 1.96, 95% CI: 1.08–3.57) and older age (OR: 1.05, 95% CI: 1.01–1.09) and, negatively, with admission in ICU (OR: 0.22, 95% CI: 0.05–0.90). In contrast, none of the objective neuropsychological test scores was significantly associated with cognitive complaints. Exploratory analysis showed that cognitive complaints were associated with both anxiety and depressive symptoms.

**Discussion:** These preliminary results suggest that cognitive complaints at 1 month after a hospitalization for COVID-19 are associated with psychological distress, independently of objective neuropsychological status. Anxiety and depression symptoms should be systematically screened in patients presenting with cognitive complaints after a severe COVID-19 episode.

## Introduction

Severe acute respiratory syndrome coronavirus 2 (SARS-CoV-2) has provoked an unprecedented health and social crisis around the world (1). This virus causes a respiratory illness named coronavirus disease 2019 (COVID-19) with classical respiratory symptoms, but also potential long-term pulmonary, cardiovascular, and neuropsychiatric complications (2). Coronaviruses such as SARS-CoV-2 are neurotropic and may also be involved in multifocal brain dysfunctions, meningitis, demyelinating encephalopathy, and strokes (3–6). One study reported that 31% of patients exhibited non-specific neuropsychiatric symptoms in the acute phase of COVID-19 infection (7), and further studies reported a range of symptoms such as anosmia, agueusia, dizziness, disorientation, and lack of attention (8–10).

Some preliminary evidence also suggests that neurocognitive sequelae might last after infection recovery, predominantly in attention, memory encoding, executive function, praxis abilities, and verbal fluency (11–14). A previous study has shown that 59% of patients had neurocognitive impairment in at least one function, 38% in immediate verbal memory and learning, 12% in delayed verbal memory, 35% in verbal fluency, and 6% in working memory (15). Beside these objective symptoms, subjective cognitive complaints seem to be among the most frequent symptoms reported by patients after virus clearance (16).

Preliminary reports generally focused on acute neurocognitive complications, but the associations between neuropsychiatric symptoms and persistent cognitive complaints after COVID-19 have been less explored (17). The association between anxiety and depression symptoms and cognitive functioning is well-established in both patients with mental disorders (18) and the general population (19). Cognitive complaints are among the core diagnostic criteria of several mental disorders, including major depressive disorder. Cognitive complaints could thus be associated with non-specific psychiatric symptoms identified after COVID-19, particularly anxiety and depressive symptoms. However, few studies simultaneously assessed anxiety and depressive symptoms and neurocognitive functioning with both subjective and objective measures among COVID 19 survivors.

In this cross-sectional study, we aimed to explore the associations between persistent cognitive complaints, objective cognitive performances (using validated tests) and symptoms of anxiety and depression. Since there are validated therapeutic interventions for both cognitive deterioration and psychological distress (12), our results could have immediate clinical implications by identifying potential clinical targets requiring early interventions.

## Materials and Methods

### Population

This study took place at the Hôpital européen Georges-Pompidou (HEGP), a French tertiary university hospital, during the first wave of the COVID-19 pandemic, between March 17th and April 29th, 2020. The study is part of The French Covid cohort study (NCT04262921) that was authorized by the French Ethics Committee (ID RCB: 2020-A00256-33). All inpatients aged 18 or more who were hospitalized at HEGP for a SARS-CoV-2 infection (according to WHO criteria) underwent a standardized clinical evaluation by a senior physician at the time of the inclusion (i.e., within the first 48 h of the hospitalization). Socio-demographic data as well as clinical and biological data were recorded at inclusion, using a standardized medical form. Clinical data included information on the initial severity of the infection such as intensive care unit (ICU) admission and the need for oxygen therapy. Biological data included the inflammatory marker C-Reactive Protein (CRP). All participants gave a written informed consent.

The patients who were discharged from hospital less than a month after the appearance of first COVID-19 symptoms were proposed a clinical follow-up at one, 3 and 6 months, either during a 1-day outpatient clinical examination or by teleconsultation. The present study used data collected during the 1-day outpatient clinical examination at 1 month.

The 1-day clinical examination included an assessment of cognitive complaints and an objective neuropsychological assessment including the Semantic Verbal Fluency Test (SVFT), the Digit Symbol Substitution Test (DSST) and the Mini Mental State Examination (MMSE). Patients also completed the Hospital Anxiety and Depression Scale (HADS) before any feedback regarding neuropsychological assessment.

Between March 17th and April 29th, 2020, 354 patients hospitalized at HEGP were included in the French Covid cohort. Among these patients, 29 were lost at follow-up because of inter-hospital transfer, 78 died and two withdrew their consent. Among the 245 patients eligible for the clinical follow-up, seven died during the follow-up, 22 withdrew their consent and 6 were not reachable. Among the 210 remaining patients who underwent the follow-up, 132 attended the 1-day follow-up examination at 1 month, including 100 who completed the neuropsychological assessment and were thus included in the present study. Table 1 displays the characteristics of the included participants.

**Table 1:** Participants characteristics (*n* = 100).

**Continuous variables**	**Median**	**iQr**
Age	60	49.5–71.5
HADS	10	6–14
MMSE (*n* = 79)	28	26–30
DSST (*n* = 97)	50	38–62
SVFT (*n* = 98)	18	14–21
C-reactive protein level at admission (*n* = 96)	90	52–147.2
**Categorical variables**	***n***	**%**
*Sex*		
Men	71	71.0
Women	29	29.0
ICU admission	31	31.0
Need for oxygen therapy at admission	73	73.0
Cognitive complaints (*n* = 100)	29	29.0
*HADS anxiety score (n = 98)*
≤ 8	67	68.4
>8; ≤ 11	17	17.3
>11	14	14.3
*HADS depression score (n = 98)*
≤ 8	76	77.5
>8; ≤ 11	14	14.3
>11	8	8.2

*HADS, Hospital Anxiety and Depression Scale; SVFT, Semantic Verbal Fluency Test; DSST, Digit Symbol Substitution Test; ICU, Intensive Care Unit; MMSE, Mini Mental Status Examination; iQr, interquartile range. The SVFT score is the number of correct generated words in 60 s and the DSST score is the number of correctly matched symbols in 120 s*.

### Cognitive Complaints Assessment

Cognitive complaints were considered present if the participant gave at least one ‘yes' answer to one of the six following questions: “Do you experience the following symptoms on a regular basis? a. forgetfulness in activities of daily living (shopping, using household appliances, etc.); b. difficulty retaining new and simple information; c. difficulty remembering old memories; d. difficulty in calculating; e. difficulty with language (finding words, recognizing objects); f. difficulty in orienting oneself in the city, in the street.” These questions were the same as those used in the French population-based CONSTANCES cohort among participants aged 45–69 (20).

### Neuropsychological Assessment

The SVFT assess lexico-semantic access and word search strategy (21). The SVFT requires participants to say as many words as possible from the “Animal” category in 60 s. The score is the number of correct generated words. The DSST is a subtest of the Wechsler Adult Intelligence Scale-Revised, a timed paper and pencil task that measures psychomotor speed, sustained attention and logical reasoning (22). At the top of the page, a coding matrix contains the digits 1–9, each of them paired with a symbol. Underneath, a series of digits with a blank space for sketching the symbol is presented. The participant has to match symbols with their corresponding numerical digit as fast as possible. The DSST score represents the number of correctly matched symbols in 120 s.

Since the cognitive profile of COVID-19 survivors was mostly unknown at the time the study was designed, the SVFT and the DSST were merely chosen for their sensitivity in detecting subtle cognitive impairment of several cognitive functions, which have eventually been reported to be impaired after COVID-19 (11–14): attention, working memory and executive functions. For instance, in the SVFT, the participant has to inhibit the words that he or she previously gave and to explore new categories of animals when a given category is no longer productive enough; in the DSST, the participant has to constantly shift between several subtasks, including checking his or her responses while learning new correspondences since more and more symbols are used as the task progresses. In addition, these tests are widely used, easy to implement and quick to perform.

The SFVT and DSST were performed in similar conditions by all participants, in a dedicated space at the beginning of the 1-day examination, under the supervision of nurses who were given written standardized instructions and trained by one of us (CL) prior to the study. The MMSE was performed under the supervision of the physician in charge of the medical examination. Since the MMSE is routinely performed by French medical students, the physician did not receive further training but was given written standardized instructions.

### Statistical Analysis

Logistic regression models were used to examine the associations between the presence of cognitive complaints and the variables of interest. Univariable analyses were first conducted, then a multivariable logistic regression model was computed to adjust for possible confounding factors, including age, sex, and admission in ICU (as a clinical marker of the initial severity of the infection). Exploratory analyses examined the HADS anxiety and depression subscales separately. As sensitivity analyses, we computed additional logistic regression models while replacing the “admission in ICU” variable with other variables representing initial severity: need for oxygen therapy or level of CRP at admission. We used MMSE in sensitivity analyzes only because of missing data. All the analyses were performed with Stata 15.0 (StataCorp, College Station, TX).

## Results

Twenty-nine participants (29%) presented with cognitive complaints at 1 month after their hospitalization for COVID-19 (Table 1).

In univariable analyses, presenting with cognitive complaints was associated with higher HADS score and older age and, negatively, with admission in ICU (Table 2). Sex was not significantly associated with cognitive complaint but was retained as an adjustment variable in the multivariable models, considering its well-known associations with both anxiety and depression and severity of SARS-CoV-2 infection.

**Table 2:** Variables associated with cognitive complaints at 1-month follow-up in logistic regression models.

	**Univariable analyses**		**Multivariable analysis (** ***n*** **=** **96)**	
	**Crude OR (CI 95%)**	***p***	**Adjusted OR (CI 95%)**	***p***
HADS	**1.94 (1.15**–**3.27)**	**0.014**	**1.96 (1.08**–**3.57)**	**0.028**
Age	**1.04 (1.01**–**1.08)**	**0.015**	**1.05 (1.01**–**1.09)**	**0.026**
Female sex	1.80 (0.72**–**4.52)	0.211	0.99 (0.33**–**3.05)	0.99
ICU admission	**0.18 (0.05-0.64)**	**0.008**	**0.22 (0.05-0.90)**	**0.035**
SVFT	0.95 (0.88**–**1.02)	0.176	0.93 (0.84**–**1.03)	0.152
DSST	0.98 (0.96**–**1.01)	0.163	1.01 (0.97**–**1.05)	0.611

*OR, odd ratio; CI, Confidence interval; HADS, Hospital Anxiety and Depression Scale; SVFT, Semantic Verbal Fluency Test; DSST, Digit Symbol Substitution Test; ICU, Intensive Care Unit. Odds ratios for HADS are given for an increment of one interquartile range (iQr). The SVFT score is the number of correct generated words in 60 s and the DSST score is the number of correctly matched symbols in 120 s. Bold values indicate statistically significant associations*.

In multivariable analyses, the association between cognitive complaints and higher HADS score persisted, with a similar odd ratio (Table 2). The other associations observed in univariable analyses also persisted. None of the objective neuropsychological test scores was significantly associated with cognitive complaints. Exploratory analyses found similar odds ratios for both the anxiety and depression subscores of the HADS. In sensitivity analyses, the observed associations and their odds ratios did not substantially change when replacing the “ICU admission” variable by another marker of severity (i.e., need for oxygen therapy or CRP level at admission) or when including the MMSE score.

## Discussion

This study investigated the association between psychological distress, objective neurocognitive functioning, and subjective cognitive complaints in COVID-19 survivors 1 month after their hospitalization. We found a robust association between cognitive complaints and psychological distress. This association was independent of objective neurocognitive functioning, which was not a significant predictor of cognitive complaints or psychological distress (data not shown). These results mainly suggest that anxiety and depressive symptoms should be assessed in patients with cognitive complaints after a severe episode of COVID-19, in order to accurately manage these symptoms.

We found a negative association between ICU admission and cognitive complaints. The length of stay in ICU is a risk factor of developing medical, neurocognitive and psychological complications (23). Objective cognitive disabilities (as measured with the MMSE) have also been associated with the length of stay in ICU in a case series of nine patients with COVID-19 (12). Regarding our results, we can postulate that patients who received ICU care were less concerned by subjective cognitive complaints than other potential physical sequelae (2). Interestingly, recent findings also suggest that the risk of persistent symptoms after COVID-19 is not associated with initial severity (24).

The main strength of our study is to have simultaneously assessed psychological distress, objective neuropsychological functioning and subjective cognitive complaints. Major depressive disorder is associated with objective neurocognitive deficits, and the cognitive complaints found in our sample could have been mediated by this objective cognitive impairment (18). However, our results showed that the association between psychological distress and cognitive complaints remained significant after adjustment for neurocognitive functioning assessed with well-established neuropsychological tests. Lastly, we used two tests (DSST, SVFT) that are sensitive to detect subtle changes in cognitive functioning (21, 25), contrasting with the MMSE that rather detects severe cognitive dysfunctions (26), such as those found in neurodegenerative diseases. Our study has several limitations: the monocentric recruitment, the absence of follow-up after 1 month, the absence of data on medication use or history of mental disorders. The small sample size also restricted the number of variables to be included in the models and the fact that only 28% of the patients hospitalized for COVID-19 were finally included in the study suggests that selection biases might have occurred. For instance, the exclusion of patients still hospitalized at 1 month is a significant selection bias. However, the rate of cognitive complaints in our sample (i.e., 29.0%) was strikingly similar to the rate observed in the French population-based CONSTANCES cohort with the same questions (i.e., 29.8% among 90,646 participants aged 45–69). Furthermore, although we chose the DSST and SFVT for their sensitivity at a time when the cognitive profile of COVID-19 survivors was mostly unknown, they may lack specificity in terms of the cognitive functions tested. Other simple cognitive tests could have more specifically assessed working memory or attention.

## Conclusion

Despite the limitations of our study, and the need to be replicated, our results suggest that cognitive complaints after a severe episode of COVID-19 should be considered as potentially signaling anxiety or depression symptoms that should be searched for and managed.

### The French COVID Study Group

Laurent ABEL (Inserm UMR 1163, Paris, France), Claire ANDREJAK (CHU Amiens, France), François ANGOULVANT (Hôpital Necker, Paris, France), Delphine BACHELET (Hôpital Bichat, Paris, France), Marie BARTOLI (ANRS, Paris, France), Romain BASMACI (Hôpital Louis Mourier, Colombes, France), Sylvie BEHILILL (Pasteur Institute, Paris, France), Marine BELUZE (F-CRIN Partners Platform, Paris, France), Dehbia BENKERROU (Inserm UMR 1136, Paris, France), Krishna BHAVSAR (Hôpital Bichat, Paris, France), Lila BOUADMA (Hôpital Bichat, Paris, France), Maude BOUSCAMBERT (Inserm UMR 1111, Lyon, France), Minerva CERVANTES-GONZALEZ (Hôpital Bichat, Paris, France), Anissa CHAIR (Hôpital Bichat, Paris, France), Catherine CHIROUZE (CHRU Jean Minjoz, Besançon, France), Alexandra COELHO (Inserm UMR 1018, Paris, France), Sandrine COUFFIN-CADIERGUES (Inserm sponsor, Paris, France), Camille COUFFIGNAL (Hôpital Bichat, Paris, France), Eric d'ORTENZIO (ANRS, Paris, France), Marie-Pierre DEBRAY (Hôpital Bichat, Paris, France), Dominique DEPLANQUE (Hôpital Calmette, Lille, France), Diane DESCAMPS (Hôpital Bichat, Paris, France), Mathilde DESVALLÉE (Inserm UMR 1219, Bordeaux, France), Alpha DIALLO (ANRS, Paris, France), Alphonsine DIOUF (Inserm UMR 1018, Paris, France), Céline DORIVAL (Inserm UMR 1136, Paris, France), François DUBOS (CHU Lille, France), Xavier DUVAL (Hôpital Bichat, Paris, France), Philippine ELOY (Hôpital Bichat, Paris, France), Vincent ENOUF (Pasteur Institute, Paris, France), Hélène ESPEROU (Inserm sponsor, Paris, France), Marina ESPOSITO-FARESE (Hôpital Bichat, Paris, France), Manuel ETIENNE (CHU Rouen, France), Nathalie GAULT (Hôpital Bichat, Paris, France), Alexandre GAYMARD (Inserm UMR 1111, Lyon, France), Jade GHOSN (Hôpital Bichat, Paris, France), Tristan GIGANTE (F-CRIN INI-CRCT, Nancy, France), Morgane GILG (F-CRIN INI-CRCT, Nancy, France), Jérémie GUEDJ (Inserm UMR 1137, Paris, France), Alexandre HOCTIN (Inserm UMR 1018, Paris, France), Ikram HOUAS (Inserm sponsor, Paris, France), Isabelle HOFFMANN (Hôpital Bichat, Paris, France), Jean-Sébastien HULOT (Hôpital Européen Georges Pompidou, Paris, France), Salma JAAFOURA (Inserm sponsor, Paris, France), Ouifiya KAFIF (Hôpital Bichat, Paris, France), Florentia KAGUELIDOU (Hôpital Robert Debré, Paris, France), Sabrina KALI (Hôpital Bichat, Paris, France), Antoine KHALIL (Hôpital Bichat, Paris, France), Coralie KHAN (Inserm UMR 1219, Bordeaux, France), Cédric LAOUÉNAN (Hôpital Bichat, Paris, France), Samira LARIBI (Hôpital Bichat, Paris, France), Minh LE (Hôpital Bichat, Paris, France), Quentin LE HINGRAT (Hôpital Bichat, Paris, France), Hervé LE NAGARD (Inserm UMR 1137, Paris, France), Soizic LE MESTRE (ANRS, Paris, France), François-Xavier LESCURE (Hôpital Bichat, Paris, France), Sophie LETROU (Hôpital Bichat, Paris, France), Yves LEVY (Vaccine Research Insitute (VRI), Inserm UMR 955, Créteil, France), Bruno LINA (Inserm UMR 1111, Lyon, France), Guillaume LINGAS (Inserm UMR 1137, Paris, France), Jean Christophe LUCET (Hôpital Bichat, Paris, France), Denis MALVY (CHU Bordeaux, France), Marina MAMBERT (Inserm UMR 1018, Paris, France), France MENTRÉ (Hôpital Bichat, Paris, France), Christelle PAUL (ANRS, Paris, France), Amina MEZIANE (Inserm UMR 1136, Paris, France), Hugo MOUQUET (Pasteur Institute, Paris, France), Jimmy Mullaert (Hôpital Bichat, Paris, France), Nadège NEANT (Inserm UMR 1137, Paris, France), Marion NORET (RENARCI, Annecy, France), Huong PHAM (Hôpital Bichat, Paris, France), Aurélie PAPADOPOULOS (Inserm sponsor, Paris, France), Nathan PEIFFER-SMADJA (Hôpital Bichat, Paris, France), Ventzislava PETROV-SANCHEZ (ANRS, Paris, France), Gilles PEYTAVIN (Hôpital Bichat, Paris, France), Valentine PIQUARD (Hôpital Bichat, Paris, France), Olivier PICONE (Hôpital Louis Mourier, Colombes, France), Oriane PUÉCHAL (REACTing, Paris, France), Manuel ROSA-CALATRAVA (Inserm UMR 1111, Lyon, France), Bénédicte ROSSIGNOL (F-CRIN INI-CRCT, Nancy, France), Patrick ROSSIGNOL (CHU Nancy, France), Carine ROY (Hôpital Bichat, Paris, France), Marion SCHNEIDER (Hôpital Bichat, Paris, France), Richa SU (Hôpital Bichat, Paris, France), Coralie TARDIVON (Hôpital Bichat, Paris, France), Marie-Capucine TELLIER (Hôpital Bichat, Paris, France), François TÉOULÉ (Inserm UMR 1136, Paris, France), Olivier TERRIER (Inserm UMR 1111, Lyon, France), Jean-François TIMSIT (Hôpital Bichat, Paris, France), Christelle TUAL (Inserm CIC-1414, Rennes, France), Sarah TUBIANA (Hôpital Bichat, Paris, France), Sylvie VAN DER WERF (Pasteur Institute, Paris, France), Noémie VANEL (Hôpital la Timone, Marseille, France), Aurélie VEISLINGER (Inserm CIC-1414, Rennes, France), Benoit VISSEAUX (Hôpital Bichat, Paris, France), Aurélie WIEDEMANN (Vaccine Research Insitute (VRI), Inserm UMR 955, Créteil, France), Yazdan YAZDANPANAH (Hôpital Bichat, Paris, France).

## Data Availability Statement

The raw data supporting the conclusions of this article will be made available by the authors, without undue reservation.

## Ethics Statement

This study involving human participants was reviewed and approved by the French Ethics Committee (ID RCB: 2020-A00256-33). The patients/participants provided their written informed consent to participate in this study.

## Author Contributions

CG, HB, and CL contributed to conception, design of the study, and wrote sections of the manuscript. CG performed the statistical analysis. HB wrote the first draft of the manuscript. All authors contributed to manuscript revision, read, and approved the submitted version.

## Conflict of Interest

CL reports personal fees and non-financial support from Janssen-Cilag, Lundbeck, Otsuka Pharmaceutical, and Boehringer Ingelheim in the previous three years, outside the submitted work. The APHP, which employs J-SH, has received research grants from Bioserenity, Sanofi, Servier and Novo Nordisk. J-SH has received speaker, advisory board or consultancy fees from Amgen, Astra Zeneca, Bayer, Bristol-Myers Squibb, Novartis, WeHealth. The remaining authors declare that the research was conducted in the absence of any commercial or financial relationships that could be construed as a potential conflict of interest.

## Publisher's Note

All claims expressed in this article are solely those of the authors and do not necessarily represent those of their affiliated organizations, or those of the publisher, the editors and the reviewers. Any product that may be evaluated in this article, or claim that may be made by its manufacturer, is not guaranteed or endorsed by the publisher.
